# Disparities in Health Insurance and Delayed Care Among Hispanic Immigrants in the United States

**DOI:** 10.7759/cureus.94294

**Published:** 2025-10-10

**Authors:** Temitope Ayodele, Connor Fletcher, Kirk B Beredjiklian, Michael Valenzuela, Zane Mazur

**Affiliations:** 1 Research, Zucker School of Medicine, Uniondale, USA; 2 Research, Rutgers New Jersey Medical School, New Brunswick, USA; 3 Research, Episcopal Academy, Newtown Square, USA; 4 Orthopaedics, Philadelphia College of Osteopathic Medicine, Philadelphia, USA; 5 Research, Hospital of the University of Pennsylvania, Philadelphia, USA

**Keywords:** delayed care, disparity, health insurance, hispanic, immigrants

## Abstract

Background: Hispanic immigrants in the United States face persistent barriers to healthcare, including lower rates of insurance coverage and a greater likelihood of delaying care due to cost. Clarifying the independent effects of ethnicity and nativity on these disparities is essential to inform effective policy.

Methods: We analyzed pooled data from the 2019-2023 National Health Interview Survey (NHIS), accessed through the Integrated Public Use Microdata Series (IPUMS). Primary outcomes were being uninsured and delayed medical care due to cost in the past 12 months. Key independent variables were Hispanic ethnicity and nativity (US-born vs. foreign-born). Covariates included survey year, age, sex, educational attainment, and family income relative to the federal poverty threshold. Insurance status was also included in the delayed care model. We used multivariable logistic regression with survey weights and design variables to estimate adjusted associations.

Results: Both Hispanic ethnicity and foreign-born status were significantly associated with higher odds of being uninsured, even after adjusting for demographic and socioeconomic factors. In the delayed care model, lack of insurance was the strongest predictor; however, Hispanic ethnicity and foreign-born status remained independently associated with increased risk. Higher education and a greater income-to-poverty ratio were protective in both models. Despite modest reductions in overall uninsurance and cost-related delays from 2019 to 2023, disparities persisted.

Conclusion: Hispanic immigrants continue to face disproportionate challenges in healthcare access, including higher uninsurance rates and cost-related care delays. While insurance status is a key determinant, disparities remain even after accounting for socioeconomic factors. Policies aimed at expanding affordable coverage and addressing systemic barriers to care for immigrant populations are needed.

## Introduction

Access to healthcare remains a persistent challenge for Hispanic and immigrant populations in the United States. Although Hispanics represent the largest minority group in the country, they continue to face disproportionately high rates of uninsurance and financial barriers to care [1,2]. Immigrants, especially those of Hispanic origin, are at increased risk of being uninsured due to limited eligibility for public insurance programs, lack of employer-sponsored coverage, and language barriers, among other concerns [3].

While legislative changes such as the Affordable Care Act (ACA) have helped reduce overall uninsurance rates, disparities in healthcare access persist [4,5]. Previous data suggest that Hispanic immigrants are less likely to have access to healthcare services than both US-born Hispanics and non-Hispanic populations, and they are more likely to delay or forgo medical care [4-6]. These inequities contribute to adverse health outcomes and further exacerbate existing health disparities. Understanding the independent and combined effects of ethnicity and nativity on access to care is crucial in informing policy interventions [7,8].

The National Health Interview Survey (NHIS) provides nationally representative data that can be used to examine healthcare access across diverse demographic groups [9]. Using pooled NHIS data from 2019 to 2023, we investigated the associations of Hispanic ethnicity and immigrant status with two key outcomes: (1) lack of health insurance and (2) delayed medical care due to cost. By analyzing these outcomes and adjusting for relevant demographic and economic factors, this study aimed to assess the persistence of disparities and to elucidate the role of insurance coverage in mediating access to care among other covariates.

## Materials and methods

We conducted a pooled cross-sectional analysis using data from the 2019-2023 NHIS, accessed via the Integrated Public Use Microdata Series (IPUMS) platform. The NHIS is a nationally representative household survey of the civilian, noninstitutionalized US population, employing a multistage, stratified probability sampling design. The analytic sample included adult respondents with complete data on health insurance coverage, nativity, and all covariates. To ensure nationally representative estimates and account for the survey design, all analyses incorporated sampling weights, strata, and primary sampling units (PSUs) provided by the NHIS.

We examined two primary outcomes related to healthcare access: (1) lack of health insurance (variable - HINOTCOV), defined as having no coverage at the time of interview, excluding single-service plans; and (2) delayed medical care due to cost (variable - DELAYCOST), defined as reporting a delay in receiving needed medical care in the past 12 months because of cost. Independent variables included (1) Hispanic ethnicity (variable - HISPETH), categorized as Mexican, Puerto Rican, Cuban, other Hispanic, and non-Hispanic (reference group); and (2) Nativity (variable - USBORN), classified as US-born versus foreign-born. Multivariable models adjusted for a range of sociodemographic and socioeconomic covariates known to influence healthcare access: (1) survey year (YEAR, 2019-2023); (2) age (AGE, continuous); (3) sex (SEX, male vs. female); (4) educational attainment (EDUC, categorical); and (5) family income relative to the federal poverty level (POVERTY, ratio measure). In the delayed care model, insurance status (variable - HINOTCOV) was included as an additional covariate to account for its mediating role in cost-related access barriers.

Descriptive statistics were used to characterize uninsured status and delayed care across ethnicity and nativity groups. Logistic regression models were then fitted to estimate adjusted associations. In Model 1, the outcome was lack of insurance (HINOTCOV). In Model 2, the outcome was delayed medical care due to cost (DELAYCOST), with insurance status included as a covariate. All analyses incorporated NHIS-provided sampling weights (SAMPWEIGHT), strata (STRATA), and primary sample units (PSU) to account for the survey’s design. Results are presented as means with corresponding 95% confidence intervals (CIs), chi-square statistics, standard errors, and p-values. Statistical significance was defined as p < 0.05. Analyses were conducted using the Survey Documentation and Analysis (SDA) 4.1.4 system hosted by IPUMS NHIS.

## Results

Between 2019 and 2023, Hispanic respondents, especially those who were born outside of the United States, consistently reported higher rates of uninsurance and delays in medical care due to cost compared to non-Hispanic respondents. Lack of insurance was most prevalent among foreign-born Hispanics, while US-born non-Hispanics had the lowest rates of coverage gaps across all survey years. A similar pattern was observed for delayed care. Foreign-born Hispanics were disproportionately affected by cost-related access barriers.

Predictors of uninsurance

In the adjusted logistic regression model examining the lack of health insurance (Table 1), both Hispanic ethnicity and foreign-born status were independently associated with significantly higher odds of being uninsured. Hispanic ethnicity was associated with increased odds of uninsurance (B = 0.002; p < 0.001) relative to non-Hispanic respondents. Foreign-born status was also independently associated with higher uninsurance risk (B = 0.001; p < 0.001). Survey year was inversely associated with uninsurance (B = -0.004; p < 0.001), indicating modest improvements in coverage over time.

**Table 1:** Logistic regression predicting the lack of health insurance Multiple R = 0.131; R² = 0.017; Wald χ² = 3,285; p < 0.001

Predictor	B (SE)	95% CI (B)	p-value	Interpretation
Hispanic ethnicity	0.002 (0.000)	0.002 – 0.002	<0.001	↑ uninsured
Foreign-born (USBORN)	0.001 (0.000)	0.000 – 0.001	<0.001	↑ uninsured
Survey year	-0.004 (0.001)	-0.005 – -0.002	<0.001	↓ uninsured
Age	0.000 (0.000)	-0.002 – 0.007	0.312	NS
Female sex	-0.026 (0.002)	-0.030 – -0.023	<0.001	↓ uninsured
Educational attainment	0.000 (0.000)	0.000 – 0.000	<0.001	↓ uninsured
Poverty ratio	-0.005 (0.000)	-0.006 – -0.005	<0.001	↓ uninsured
Constant	8.733 (1.346)	6.095 – 11.370	<0.001	—

Protective factors included female sex (B = -0.026; p < 0.001), higher educational attainment (B = 0.000; p < 0.001), and higher income-to-poverty ratio (B = -0.005; p < 0.001). Age was not significantly associated with uninsurance. The model was statistically significant overall (Wald χ² = 3,285; p < 0.001), although explanatory power was limited (R² = 0.017), suggesting that uninsurance is influenced by a broad range of structural and individual factors.

Predictors of delayed care due to cost

In the second regression model (Table 2), which assessed delayed medical care due to cost, both Hispanic ethnicity and foreign-born status remained significant predictors after adjusting for sociodemographic characteristics and insurance status. Hispanic ethnicity (B = 0.001; p < 0.001) and foreign-born status (B = 0.016; p < 0.001) were associated with higher odds of delaying care. Lack of insurance emerged as the strongest predictor in the model (B = 0.157; p < 0.001), with uninsured individuals substantially more likely to report cost-related delays. Additional risk factors included older age (B = 0.000; p < 0.001) and female sex (B = 0.016; p < 0.001).

**Table 2:** Logistic regression predicting delayed medical care due to cost (National Health Interview Survey (NHIS) 2019-2023) Multiple R = 0.394; R² = 0.155; Wald χ² = 34,592; p < 0.001

Predictor	B (SE)	95% CI (B)	p-value	Interpretation
Hispanic ethnicity	0.001 (0.000)	0.001 – 0.001	< 0.001	↑ delayed care
Foreign-born (USBORN)	0.016 (0.000)	0.016 – 0.016	< 0.001	↑ delayed care
Survey year	-0.005 (0.001)	-0.007 – -0.003	< 0.001	↓ delayed care
Age	0.000 (0.000)	0.000 – 0.000	< 0.001	↑ delayed care
Female sex	0.016 (0.003)	0.011 – 0.021	< 0.001	↑ delayed care
Educational attainment	0.000 (0.000)	0.000 – 0.000	< 0.001	↓ delayed care
Poverty ratio	-0.003 (0.000)	-0.004 – -0.003	< 0.001	↓ delayed care
No insurance (HINOTCOV)	0.157 (0.003)	0.151 – 0.164	< 0.001	Strongest predictor of delay
Constant	10.506 (1.847)	6.886 – 14.125	< 0.001	—

Protective factors included higher educational attainment (B = 0.000; p < 0.001) and higher income-to-poverty ratio (B = -0.003; p < 0.001). Survey year again showed a small negative association (B = -0.005; p < 0.001), reflecting a modest decline in cost-related care delays over the study period. This model demonstrated stronger explanatory capacity than the uninsurance model (Wald χ² = 34,592; p < 0.001; R² = 0.155), underscoring the significant role of insurance coverage and socioeconomic factors in shaping access to care.

Table 3 denotes health insurance status between US-born non-Hispanics, US-born Hispanics, and foreign-born Hispanics. Foreign-born Hispanics have the highest uninsured rate (~26%), much greater than US-born Hispanics (~8%) and non-Hispanics (~4%). Similarly, Table 4 notes differences in delaying care due to cost among these groups. The differences are also statistically significant. Foreign-born Hispanics were nearly twice as likely to delay care due to cost compared with the other groups (p < 0.001).

**Table 3:** Health insurance status χ²(2) = 8659.5, p < 0.0001

Group	Insured	Uninsured	% Uninsured
Hispanic, foreign-born	7,449	2,683	26.50%
Hispanic, born in the US	17,092	1,443	7.80%
Non-Hispanic, born in the US	133,577	5,755	4.10%

**Table 4:** Delayed care due to cost χ²(2) = 493.3, p < 0.0001

Group	No Delay	Delay	% Delayed
Hispanic, foreign-born	9,037	1,099	10.80%
Hispanic, born in the US	17,525	1,029	5.60%
Non-Hispanic, born in the US	131,707	7,673	5.50%

## Discussion

This study highlights ongoing disparities in healthcare access among Hispanic and immigrant populations in the United States. Using nationally representative data from the 2019-2023 NHIS, we found that both Hispanic ethnicity and foreign-born status were independently associated with higher odds of being uninsured and medical care being delayed due to cost. These disparities remained significant after adjusting for social, demographic, and economic covariates, suggesting that barriers to healthcare access extend beyond individual characteristics, such as income or education. Insurance coverage was the largest disparity driver. Nearly one in four foreign-born Hispanics is uninsured, compared with fewer than one in 10 US-born Hispanics. Delays due to cost parallel insurance gaps, with foreign-born Hispanics reporting the highest care delay rates.

Consistent with prior studies, foreign-born Hispanic individuals exhibited the highest rates of uninsured status and cost-related delays in care [2,5]. Although national insurance coverage expanded following implementation of the Affordable Care Act (ACA), our findings suggest that these gains were not equitably distributed [8-11]. Insurance status was the strongest predictor of delayed care due to cost, yet the continued significance of ethnicity and nativity (even after adjusting for insurance) suggests the presence of additional structural barriers. These may include limited provider availability, language differences, fear of interacting with public systems, or experiences of discrimination, all of which can reduce access even when coverage is present [12-15].

Notably, the regression model predicting delayed care due to cost demonstrated substantially greater explanatory power (R² = 0.155) than the model predicting uninsured status (R² = 0.017). This suggests that variables such as insurance status, education, and income were more effective at explaining financial barriers to care compared to insurance coverage alone. The modest R² value in the lack of insurance model may reflect the influence of policy-level and structural factors not captured in the individual survey data. The persistent effects of Hispanic ethnicity and foreign-born status in both models also point to underlying inequities not explained by the examined covariates.

The different roles of age across the two models are also notable. Age was not a statistically significant predictor of uninsured status, suggesting no clear pattern of insurance coverage across the adult lifespan after controlling for other factors. This may reflect competing dynamics: younger adults often experience gaps in employer-based coverage, while older adults are more likely to gain coverage through Medicare [16,17]. In contrast, age was modestly but significantly associated with delayed care due to cost, implying that financial barriers may increase with age even among the insured. This could be due to higher healthcare utilization, greater out-of-pocket expenses, or fixed incomes among older adults. These findings highlight the importance of not only expanding insurance coverage but also improving the financial adequacy of that coverage, particularly for older populations.

While most social and demographic variables behaved as expected, the consistent protective effects of higher education and income-to-poverty ratio across both models reinforce the critical role of socioeconomic status in shaping healthcare access. However, the persistence of disparities after adjusting for these variables suggests that improving individual socioeconomic position alone is unlikely to eliminate inequities without addressing broader systemic issues [1,3,7,12]. The combination of Hispanic ethnicity and immigrant status likely compounds vulnerability to healthcare exclusion [2,5]. Foreign-born Hispanics often face marginalization from immigration-related exclusions and also possibly from structural racism that may affect both access to coverage and willingness to seek care [7-12].

This study has several strengths, including the use of recent, nationally representative NHIS data and the pooling of five years of data to increase statistical power. However, several limitations should be acknowledged. The cross-sectional design precludes establishing causal relationships. Self-reported measures may be subject to recall or social desirability bias, particularly for sensitive topics such as insurance coverage or healthcare utilization. In addition, the NHIS does not collect information on immigration documentation status, duration of residence, or preferred language - factors that may influence healthcare access. It is possible that documentation of immigration status (citizenship, permanent resident) would afford the ability to provide further delineation as to whether these factors contribute to insurance status. Finally, while statistically significant, some model coefficients were small in magnitude, may not be clinically significant, and reflect the complexity of factors shaping health disparities.

Future research should incorporate more specific measures of immigration-related variables, such as visa type and legal status, given that naturalized citizens have higher rates of health insurance coverage [18]. Longitudinal data would allow us to assess how changes in state or federal policy environments affect access to care over time. Additionally, qualitative studies could provide important context to better understand the factors underlying these differences. A deeper understanding of how ethnicity, nativity, and insurance status interact over time is essential for designing interventions that not only increase coverage but also promote equitable, timely, and appropriate care across different communities.

## Conclusions

Hispanic immigrants in the United States remain disproportionately more likely to be uninsured and to delay medical care due to cost. While lack of health insurance is the strongest predictor of cost-related access barriers, disparities persist even after accounting for coverage and socioeconomic factors. These findings highlight the need for policies that expand insurance coverage and address structural barriers to care for immigrant communities.
